# Bacteria

**Published:** 1885-11

**Authors:** 


### HALL'S JOURNAL OF HEALTH

Vol. 32. NOVEMBER, 1885. No. 11.

### Bacteria.

About two hundred years ago Leewenhock discovered in the tartar of his own teeth a multitude of little active beings. He expressed his surprise that the animalculæ in his mouth should exceed in number the entire population of the kingdom.

Similar organisms were found later in stagnant water, and in decaying vegetable and animal matters. Further investigation also showed that they were plants and not animals.

Many of these organisms are in the form of siiff, or flexible rods, two to twenty times as long as wide, and hence the name Bacteria was applied to them.

Bacteria belongs to the lowest family of plants, being composed of a single cell, with cellulose outer wall, and fluid protoplasmic contents They are found wherever there is decomposing organic matter. Furthermore, organic matter does not decompose, unless they be present, and hence they are regarded as the cause, or agents of putrefaction, fermentation and decay. This hypothesis has its practical demonstration in the preservation of canned fruits, vegetables and meats. They are put into the cans boiling hot, and the cans are immediately sealed. The boiling kills the bacteria, and the steam excludes most of the air Thus the development of bacteria being prevented, decomposition cannot take place, and hence the substance in the can keeps good for months, even for years.

Bacteria develop with great rapidity. They increase by simply dividing, one becoming two, and these again dividing in a short time. Prof. Cohn, of Breslau, observed that a bacterium termo would thus divide itself every hour. He calculated that in twenty-four hours the progeny of one bacterium would occupy the space of one thousandth of a cubic inch on the side. This amount would then double every hour, and continuing the calculation, he found that in five days the number would be sufficient to fill all the oceans of the globe! Of course the extent of their development is limited by the supply of food. But we have here a sufficient explanation of the rapidity with which putrefaction takes place.

Bacteria can only be seen with a good microscope. They are best studied with a magnifying power of from five hundred to one thousand diameters. Their average thickness is about one twenty-five thousandth of an inch. A thousand could swim side by side through the eye of a needle, and you could hold fifty millions of millions in the palm of your hand.

Bacteria are useful in many ways. They purify the water of rivers, reservoirs and cisterns. They rot flax and bleach cotton, they infect insects, producing a disease which destroys them by the thousand, thus ridding mankind of these pests ; they devour filth and offal, which without them would accumulate to an indefinite extent extent. Without them nothing would decay. The ground would become covered with the dead bodies of plants and animals ; no soil would be formed, vegetation would die, then animals would perish for the want of food, and earth would become a lifeless waste, covered with its inhabitants, all dead but undecayed. Thus the very habitableness of the globe is found to depend upon these, perhaps the most insignificant of God's creatures."

